# *Nicotiana tabacum* Leaf Waste: Morphological Characterization and Chemical-Functional Analysis of Extracts Obtained from Powder Leaves by Using Green Solvents

**DOI:** 10.3390/molecules28031396

**Published:** 2023-02-01

**Authors:** Mariana Leal, María Alejandra Moreno, Patricia Liliana Albornoz, María Inés Mercado, Iris Catiana Zampini, María Inés Isla

**Affiliations:** 1 Instituto de Bioprospección y Fisiología Vegetal (INBIOFIV), CONICET—Universidad Nacional de Tucumán (UNT), San Miguel de Tucumán T4000CBG, Argentina; 2 Instituto de Morfología Vegetal, Fundación M. Lillo, Miguel Lillo 251, San Miguel de Tucumán T4000JFE, Argentina; 3 Facultad de Ciencias Naturales e IML, Universidad Nacional de Tucumán (UNT), San Miguel de Tucumán T40000JFE, Argentina

**Keywords:** alkaloids, antioxidant activity, green solvent, NaDES, phenolic compounds, pre-harvest waste, tobacco

## Abstract

Tobacco cultivation and industrialization are characterized by the production of trillions of pre-harvest and post-harvest waste biomasses each year with the resulting negative effects on the environment. The leaves of blunt, pre-harvest waste, could be further used to obtain bioactive metabolites, i.e., polyphenols and alkaloids, for its potential cosmetic use. This study was conducted to obtain bio-compounds from pre-harvest tobacco leaf waste (var. Virginia) by applying conventional and green solvents (NaDES). Leaves and ground leaf waste were characterized based on their microscopic features. Conventional solvents, such as water, acetone, ethanol, and non-conventional solvents, such as Natural Deep Eutectic Solvents (NaDES), i.e., sucrose:lactic acid (LAS), frutose:glucose:sucrose (FGS), lactic acid:sucrose:water (SALA), choline chloride:urea (CU), and citric acid: propylene glycol (CAP) were used for bioactive extraction from tobacco waste powder. CU, FGS, and acetone/ethanol had similar behavior for the best extraction of alkaloids (6.37–11.23 mg ACE/g tobacco powder). LAS, FGS, SALA, and CU were more effective in phenolic compound extraction than conventional solvents (18.13–21.98 mg AGE/g tobacco powder). Because of this, LAS and SALA could be used to obtain phenolic-enriched extracts with lower alkaloid content rather than CU and FGS. Extracts of the powder obtained with conventional solvent or CU showed a high level of sugars (47 mg/g tobacco powder) The ABTS antioxidant capacity of tobacco leaf powder was higher in the extracts obtained with CU, FGS, and acetone (SC_50_ 1.6–5 µg GAE/mL) while H_2_O_2_ scavenging activity was better in the extracts obtained with LAS, CAP and SALA (SC_50_ 3.8–8.7 µg GAE/mL). Due to the biocompatibility of the NaDES with the components of tobacco leaf waste, the opportunity to apply these extracts directly in antioxidant formulations, such as cosmetics, phytotherapic, and other formulations of topic use seems promising. Furthermore, NaDES constituents, i.e., urea and organic acid can also have beneficial effects on the skin.

## 1. Introduction

*Nicotiana tabacum* L., or tobacco, belongs to the family Solanaceae Juss. It is an annual or perennial, adventitious herb that grows between 0 and 500 m above sea level (m.a.s.l) [1].

As a smokeable product, tobacco is one of the most important lines in the economies of many countries; seven of the largest producers are countries on the American continent. In the period 2019–2020, Argentina reported a production of 106,319 tons of tobacco [2]. Six types of tobacco are produced in Argentina in the Northern provinces of Jujuy (37%), Salta (34%), Misiones (22%), Tucumán (4%), Corrientes (1%), Catamarca and Chaco (<1%); three of them are light (Virginia, Burley, and Criollo Salteño) and three are dark (Criollo Misionero, Criollo Correntino, and Kentucky) with different technical characteristics. Light tobacco, mainly Virginia and Burley, are the most important crops, accounting for more than 90% of the national production. In the period 2014–2015, the Virginia variety contributed to almost 70% of the total production of the country [3,4].

According to recent reports, the global tobacco annual production in 2020 was 5.88 million metric tons [2], and this led to trillions of waste materials each year [5] with follow-up negative effects on the environment [6].

**Figure 1:**
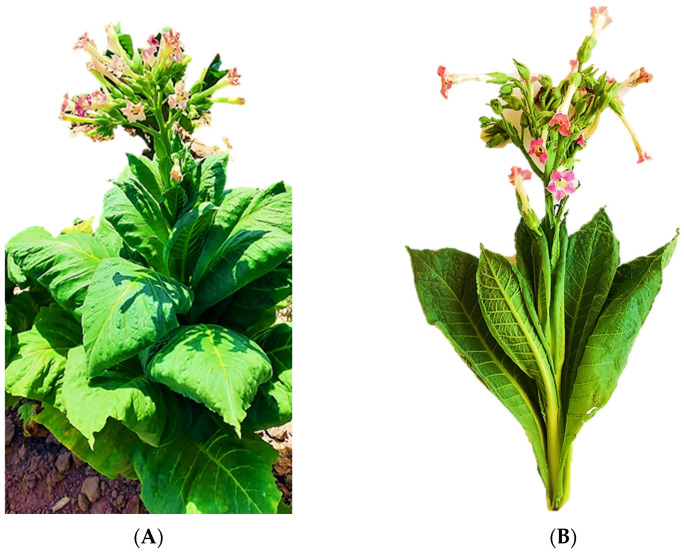
*Nicotiana tabacum* var. Virginia. (**A**) Plant with flowers prior to the blunt at the field. (**B**) General aspect of pre-harvest waste collected during the blunt (flowers and apical leaves).

The tobacco waste is accumulated during the processing of plants, pre, and post-harvest. Before harvest, some leaves and flowers are discarded in the field, following an agricultural practice named “blunt or deflowering” (Figure 1); this task consists of hand cutting the flower axes, together with some lateral buds, to prevent the production of new leaves. Better yields and good quality products ensue thanks to this practice at the end of the harvest. Moreover, during the handling in the field, some broken leaves are discarded (scrap), while the midribs, as well as the smallest pieces of tobacco waste, which do not serve any purpose (dust or offal), are removed during the manufacturing of cigarettes [5,7].

The chemical studies revealed that tobacco leaves are rich in sterols, diterpenoids, sesquiterpenes, alkaloids (including nicotine), and phenolic compounds i.e., flavonoids, phenolic acids, and coumarins [8,9,10,11,12,13,14,15,16,17]. Hence, tobacco leaves may well be an important element in the treatment of cancer, and can also act as neuroprotective, antioxidant, anti-inflammatory, antimicrobial, antitumoral, and antiparasitic among other assets [8,12,14,15,16,17,18,19,20,21,22,23] as numerous studies have shown.

Within this context, pre-harvest waste or biomass, i.e., leaves of blunt or deflowering (Figure 1), resulting from the tobacco var. Virginia could be further used as a subproduct to obtain bioactive metabolites (polyphenols and alkaloids) for future novel purposes, namely in the cosmetic industry. Currently, the worldwide trend is to reduce the consumption of cigarettes, and therefore alternative uses of tobacco and its waste are in great demand [24]. In other words, industries other than cigarette manufacturing, could be developed, and the experience gained over so many years could be a trump card for tobacco producers as new job posts would be created and the economic damage that the cessation of tobacco products causes could be changed into a profitable business so as to give a new impulse to the tobacco industry and help improve the economy of the country.

When it comes to obtaining bioactive metabolites, the efficiency of metabolite extraction from plant biomass, solubility, and stability are among the essential criteria to select the most suitable solvent to develop a successful value chain. The organic solvents are efficient but relatively toxic and could remain not only as a residual pollutant in the extracts produced but also as air pollutants that contribute to global warming [25,26]. The use of green solvents could overcome the limitations of conventional organic solvents. Natural deep eutectic solvents (NaDES) are considered “natural or green” because the constituent components are plants’ primary metabolites, i.e., sugars, organic acids and bases, and amino acids. NaDES are not volatile, non-toxic, and can extract both hydrophilic and hydrophobic molecules [27,28]. Furthermore, the NaDES showed both chemical and thermal stability; these properties are associated with the stability of the extracted bioactive molecules during storage for their subsequent incorporation into a pharmaceutical and/or cosmetic product. Moreover, different solvents/biomass ratios, different extraction conditions, and extraction methodologies could be used to obtain biomolecules, i.e., maceration, liquid biphasic flotation, ultrasound, supercritical fluids, microwave-assisted extraction [5,7,17,29,30,31,32,33].

Based on the aforementioned analysis, the present research focuses on the characterization of the anatomy of *N. tabacum* (var. Virginia) waste leaves and powders obtained from them; it also deals with optimizing the extraction of bioactive compounds by using conventional and green solvents (NaDES) and assessing their antioxidant activity. The present work reveals the effectiveness of NaDES for the selective extraction of antioxidant compounds from tobacco pre-harvest waste and supports their utilization in the cosmetic industries.

## 2. Results and Discussion

**Figure 2:**
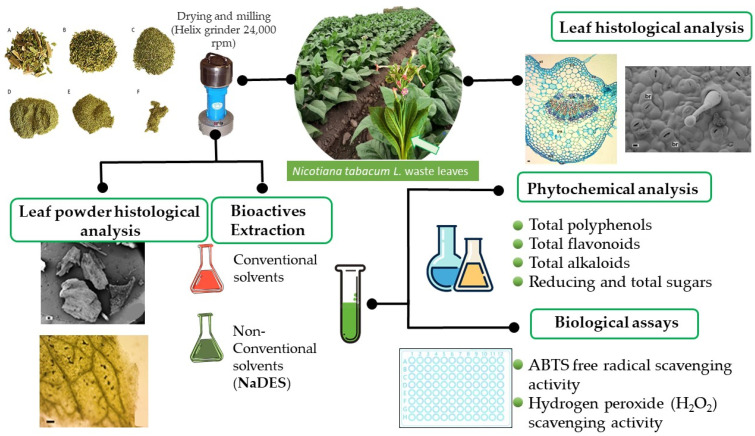
Flowchart illustrating the process of tobacco leaf waste under study.

A bioactive secondary metabolite profile is related to various factors, namely, tobacco varieties, growing regions, or fields with varying altitudes, latitudes, and ultraviolet radiation, age, and the organs analyzed [8,9,10,11,12,13,14,15,16,17,18,19,20,21,22,23]. Moreover, both the method and solvents, as well as the conditions of extraction of plant biomass play a key role in the chemical composition of extracts, and consequently in its biological activity. In the present study, leaf waste from the blunt of *Nicotiana tabacum* var. Virginia was collected in a field in Perico, Jujuy, Argentina. Tobacco leaf waste without visible injuries was used for morphological studies. Whereas another part of the plant material was grounded to obtain leaf waste powder, which was microscopically characterized and used for extraction of bioactive compounds by using conventional and green solvents (NaDES) and for assessing their antioxidant activity (Figure 2).

### 2.1. Leaf Morphology and Anatomy

**Figure 3:**
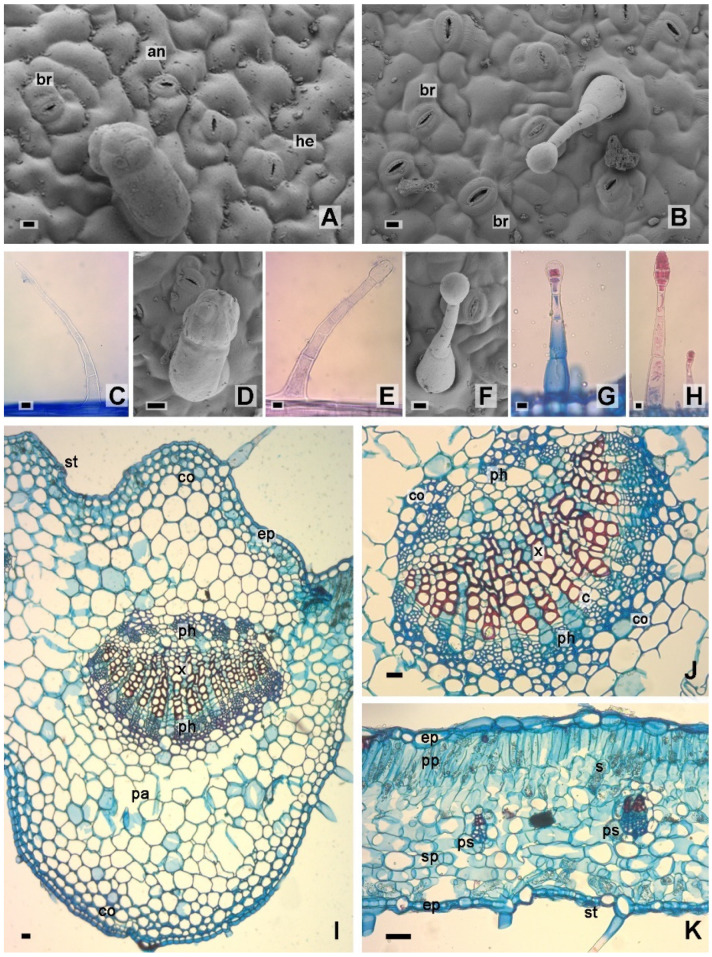
*Nicotiana tabacum* var. Virginia Leaf anatomy. (**A**,**B**). Surface view of epidermis. (**A**). Upper. (**B**). Lower. (**C**–**H**). Epidermal trichomes. (**C**). Simple trichome. (**D**). Type I glandular trichome, unicellular foot, and multicellular head. (**E**). Type IIa glandular trichome, uniseriate foot, and unicellular club-shaped head. (**F**). Type IIb glandular trichome, uniseriate foot, and rounded unicellular head. (**G**). Type IIc glandular trichome, uniseriate foot, and bicellular head. (**H**). Type IId glandular trichome, uniseriate foot, and multicellular head. (**I**–**K**). Sheet cross section. (**I**). Mid vein. (**J**). Detail of the vascular bundle of the middle vein. (**K**). Lamina. References: an, anomocytic stoma; br, brachyparacytic stoma; c, cambium; co, collenchyma; ep, epidermis; he, hemiparacytic stoma; ph, phloem; pa, parenchyma; pp, palisade parenchyma; ps, parenchymal sheath; s, starch; sp, spongy parenchyma; st, stoma; x, xylem. Scales: (**A**–**H**), 10 µm; (**I**–**K**), 20 µm.

On a superficial view, both epidermises presented rectangular to isodiametric cells with thin, wavy walls and smooth to slightly striated cuticles around the stomata. The stomata were anomocytic-, hemiparacytic-, and brachiparacytic-type on both surfaces, the latter being more scarce (Figure 3A,B). A significant statistical difference was observed in terms of the width and density of stomata, being higher in the abaxial epidermis (Table 1). Simple and glandular trichomes were found on both epidermises. The former were scarce, multicellular, and uniseriate (Figure 3C), while the glandular ones were classified into five types that can be separated into two groups: (I) unicellular foot with a multicellular head (Figure 3D) and (II) uniseriate foot with a head of different types: (IIa) club-shaped unicellular head (Figure 3E), (IIb) round unicellular head (Figure 3F), (IIc) bicellular head (Figure 3G), and (IId) multicellular head (Figure 3H). Abundant trichomes, simple as well as glandular, (ST and GTs) were found on both epidermises. The GTs are quite central in tobacco since they synthesize, store and secrete large amounts of diterpenoids, flavonoids mainly derived from methyl quercetin, and other metabolites [11,34].

In the cross section, the lamina was dorsiventral and amphistomatic; both epidermises were one-layered with thin cuticles (Figure 3I,K). The vascular bundles of the first and second order were bicollateral, while those of the lower order veins showed collateral bundled with a parenchymal sheath. Both types of vascular bundles presented collenchyma caps towards the phloem (Figure 3J,K). The palisade parenchyma presented two layers of cells, and the spongy parenchyma five to seven layers (Figure 3K). Four to six layers of subepidermal, lamellar, angular, and lacunar collenchyma were observed at the main veins (Figure 3I,J). Starch grains were evidenced mainly in the palisade parenchyma (Figure 3K).

### 2.2. Tobacco Leaf Powder Characterization

**Figure 4:**
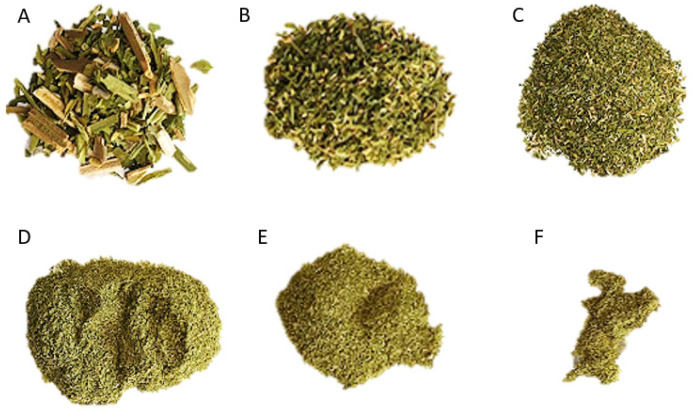
*N. tabacum* var. Virginia leaf waste powder. Sieved fractions from ground leaf waste. (**A**) 840 µm. (**B**) 500 µm. (**C**) 149 µm. (**D**) 105 µm. (**E**) 74 µm. (**F**) <74 µm.

The particle size was significantly reduced by grinding it to a micron scale and the specific surface area sharply increased. The tobacco leaf powder showed particles whose size ranged between 74 µm and 840 µm. The most abundant particle size was between 149 and 840 µm (Table 2, Figure 4).

**Figure 5:**
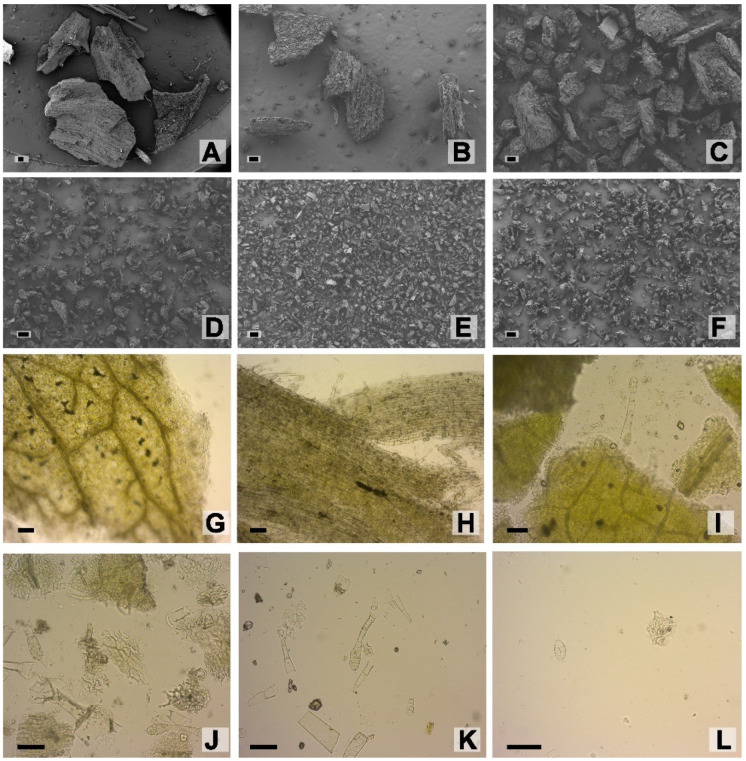
*Nicotiana tabacum* var. Virginia leaf waste powder. Sieved fractions from ground leaf waste. (**A**–**F**). SEM. (**G**–**L**). Optic microscopy. (**A**,**G**). 840 µm. (**B**,**H**). 500 µm. (**C**,**I**). 149 µm. (**D**,**J**). 105 µm. (**E**,**K**). 74 µm (**F**,**L**). <74 µm. Scales: (**A**–**L**), 100 µm.

Microscopical analyses of the sieved fractions from ground leaf waste, under scanning electron and optical microscopy, showed leaf particles of different sizes and tissue composition. The 840 µm, 500 µm, and 149 µm fractions presented complete fragments of leaves with trichomes, veins, and fibers. Instead, 105 µm, 74 µm, and <74 µm fractions exhibited mainly trichomes, trichome heads, parenchyma cells of the mesophyll, cuticle, epidermal remains and occasional fragments of xylem vessel elements (Figure 5). Grinding of dried waste leaves effectively reduced the particle sizes to the micron scale allowing an increased specific surface area for extraction trials.

### 2.3. Phytochemical Characterization by Conventional and Non-Conventional Solvents

Several authors reported different extraction methods of bioactives from tobacco and its waste [5,7,17,29,30,31,32,33]. However, no information could be found concerning the chemical composition of phenolic compounds, flavonoids, sugars, and alkaloids of tobacco leaf waste extracts; using non-conventional solvents such as NaDES does not appear to have been in the scope of any researcher. Thus, to the best of our knowledge, this is the first report on the bioactive extraction of tobacco leaf waste using NaDES.

To illustrate the potential of NaDES in the extraction of secondary and primary metabolites from ground leaf waste, comparisons of the extraction efficiency between five NaDES, i.e., LAS, SALA, CAP, FGS, CU (Table 3), and the traditional solvents AW, ethanol 70°, and water were performed. The content of total phenolic compounds (TPC) of *N. tabacum* leaf powder var. Virginia ranged from 824 to 1300 µg GAE/mL in the extraction with conventional solvents (Table 4). The content of TPC was higher in the extracts obtained with AW and ethanol 70° than in those with water. The same behavior was observed for total flavonoid compounds with values between 133.6 and 151.3 µg QE/mL for AW and ethanol 70° extracts, respectively.

LAS, FGS, SALA, and CU were more effective in TPC extraction (Table 4). It is worth mentioning that the solubility increased in NaDES by 1.6 to 2.6 times compared to ethanol and water, respectively. CU was also the best solvent for flavonoid extraction with a si-milar yield as that of both ethanol 70°, and AW.

**Figure 6:**
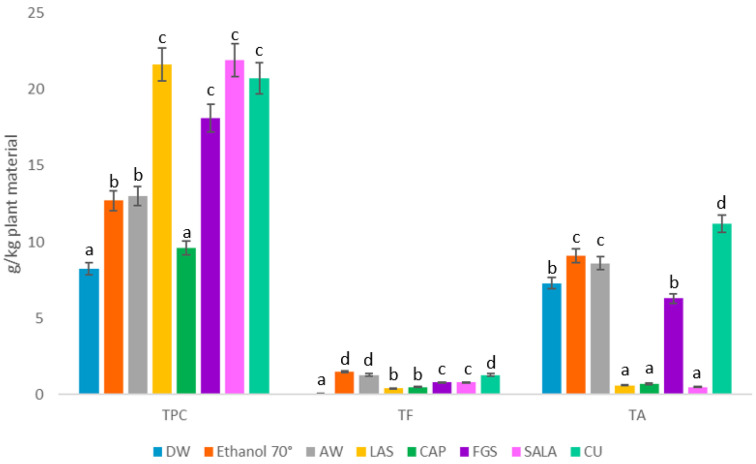
Extraction yield of total phenolic compounds (TPC); total flavonoids (TF) and total alkaloids (TA) in g/kg plant material. DW: distilled water; AW: Acetone: water; NaDES: sucrose: lactic acid: distilled water (LAS), citric acid: propylene glycol (CAP), fructose: glucose: sucrose: distilled water (FGS), sucrose: lactic acid: distilled water (SALA), choline choride: urea: distilled water (CU). Different letters in each column indicate significant differences in the TPC, TF and TA content between extracts according to Tukey’s test (*p* ≤ 0.05).

In a previous study [17], phenolic compounds were extracted from stalk and leaf waste (var. Kentuky and Burley) by using ethanol:water 50%. The phenolic compound content was 15.7 g/kg of plant material (var. Kentucky) and 12.6 g/kg of plant material (var. Burley). Similar results were obtained in this report with ethanol 70° and NaDES for leaf waste (var. Virginia) with 12 to 21.98 g/kg plant material. Figure 6 shows the yield of TPC, TF, and TA in g/kg plant material. In tobacco waste of the two varieties, Kentucky and Burley, the most abundant components reported were quinic and chlorogenic acids, rutin, and luteolin rutinoside [17]. Wang et al. [35] also reported that the dominant polyphenols in tobacco leaves were chlorogenic acid and rutin. Schwingel et al. [11] reported the presence of 3-O-methylquercetin in the ethanolic extract obtained by immersion of leaves (var. Virginia).

Regarding the extraction, the pH values of NaDES are also quite significant, CU has shown the highest pH (pH 6) [36] while the NaDES which contains organic acids such as LAS and SALA have the lowest pH values, between 1 and 4. The extraction capacities of tailor-made NaDES are highly dependent on both the physical-chemical composition of the solvent prepared and the chemical structure of the target compounds [37].

Two NaDES (CU, FGS) and conventional solvents (DW, AW, and ethanol 70°) had similar behaviors, these being the best solvents for the extraction of alkaloids from tobacco leaf powder with values between 637.2 and 1123 µg ACE/mL. Alkaloids are nitrogen-containing compounds poorly soluble in water but soluble in organic solvents. Takla et al. [38] have lengthily compared and evaluated, for the first time, the potential and effectiveness of NaDES for the extraction of Amaryllidaceae alkaloids from *Crinum powellii* Hort. bulbs. These authors demonstrated that the extraction with water-based choline chloride:fructose was significantly improved if compared to traditionally used methods that require the consumption of organic solvents and water. In the present work, NaDES such as LAS, CAP, and SALA succeeded in extracting 15 times fewer alkaloids than CU and FGS. Nevertheless, LAS, FGS, SALA, and CU were more effective in phenolic compound extraction from leaf waste powdered biomass than with conventional solvents (DW, AW, ethanol 70°). Considering that nicotine is a unique alkaloid component of both tobacco plants and other Solanaceae crops, harmful to human health, LAS and SALA NaDES may be recommended to obtain phenolic enriched extracts with lower alkaloid content, while CU and FGS solvents could be used to obtain extracts with high levels of both types of compounds.

The powder showed high levels of total and reducing sugars, both of which were extracted mainly with conventional solvent and CU.

### 2.4. Antioxidant Activity

Reactive oxygen species (ROS) are cellular metabolic products, categorized as free radicals and non-free radical species (H_2_O_2_). Oxidative stress is caused by imbalanced amounts of ROS, which damage the human body. Superoxide dismutase (SOD) has been recognized as a major component of the defense system against oxidative stress caused by ROS. In the presence of transition metal ions, it can catalyze the conversion of O_2_^•−^ to H_2_O_2_ and oxygen (O_2_), and H_2_O_2_ can be decomposed into a hydroxy radical (·OH). This radical is one of the most reactive free radicals, as it can pass through cell membranes and react with DNA, proteins, lipids, and carbohydrates [39,40].

Due to the different mechanisms of action and the nature of antioxidant substances in a complex mixture, there is no single method to determine the antioxidant power, so a proper selection of methods is required. For this reason, the antioxidant capacity of different extracts of tobacco leaf waste powders was compared by using different methodologies (ABTS and H_2_O_2_ scavenging activity). The SC_50_ values were defined as the concentration of antioxidants needed to reduce 50% of the initial free radical. A lower SC_50_ value entails a higher antiradical activity of the extract. The highest antioxidant activity on ABTS scavenging was observed for extracts obtained with CU and FGS as solvents with the lowest SC_50_ values similar to quercetin, an antioxidant flavonoid (Table 5). The effectiveness was similar to acetonic extract (SC_50_ = 5 µg GAE/mL). This enhanced ability to eliminate the ABTS radical could be related to the presence of polyphenols and alkaloids, since both compound types are extracted with these solvents [41]. The ABTS cation radical scavenging activity increased in a concentration-dependent manner due to hydrogen atom transference and single electron transference.

The extracts obtained with LAS, CAP, and SALA, three NaDES with different hydrogen bond donors, namely, citric acid, lactic acid, and propylene glycol, showed the highest H_2_O_2_ scavenging activity and reduced the generation of HO^•^ radicals in a dose-dependent manner (Table 5). This activity was significantly higher than that of the extracts obtained with conventional solvents. The experimental data evinced that some NaDES (LAS, FGS, SALA, and CU) show an excellent ability for the extraction of antioxidant compounds from tobacco leaf powder. These extracts can represent a promising material to be included in topically applied cosmetic products, as they are able to reduce oxidative stress, thus preventing cellular aging and inflammatory processes. NaDES can also have beneficial effects per se, due to their constituents. Urea, for instance, is a component of the na-tural moisturizing factor of the skin and can help preserve skin integrity and even be useful in the treatment of some skin diseases [42]. Organic acids can have numerous beneficial effects on the skin and are therefore used in the treatment of acne or as anti-aging agents for wrinkles [43].

### 2.5. Correlation between the Total Phenolic Compounds, Total Flavonoid and Total Alkaloid Content, and Antioxidant Activities

Pearson’s correlation coefficient was applied to evaluate the relationship between the antioxidant activity, i.e., ABTS and H_2_O_2_, and secondary metabolite content, including total phenolic content (TPC), total flavonoids (TF), and total alkaloids (TA), as shown in Table 6. TPC showed a significant positive correlation with ABTS radical cation scavenging activity with a Pearson’s correlation coefficient r = 0.32 (*p* < 0.05). Furthermore, significant positive correlations between both TF and TA and H_2_O_2_ scavenging activity were found with Pearson’s correlation coefficients r = 0.26 and r = 0.77, respectively (*p* < 0.05). In a previous report [17], a positive correlation between phenols of tobacco with DPPH free radical scavenging activity and a free radical with an antioxidant action mechanism similar to ABTS cation free radical was shown. Nevertheless, this is the first report on H_2_O_2_ scavenging activity and its relationship with the tobacco flavonoid and alkaloid content.

**Figure 7:**
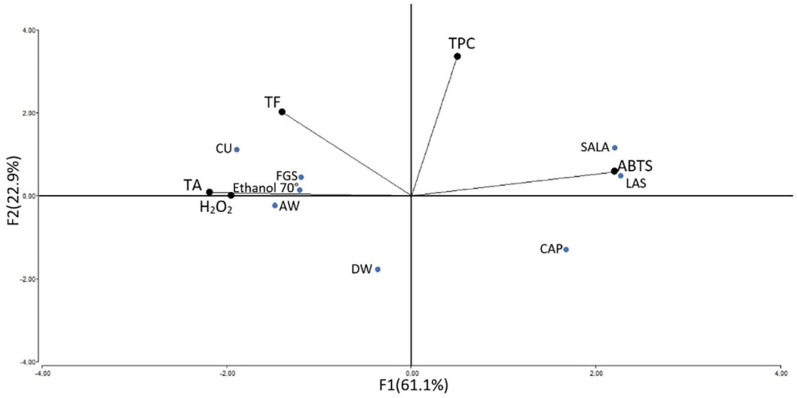
Main component analysis of the total phenolic content (TPC), total flavonoid (TF), and total alkaloids (TA) and antioxidant activity (ABTS, and H_2_O_2_ scavenging activity) of extracts from tobacco leaf waste obtained with conventional and non-conventional solvents. DW: distilled water, AW: Acetone: water, NaDES: LAS, CAP, FGS, SALA, CU.

**Figure 8:**
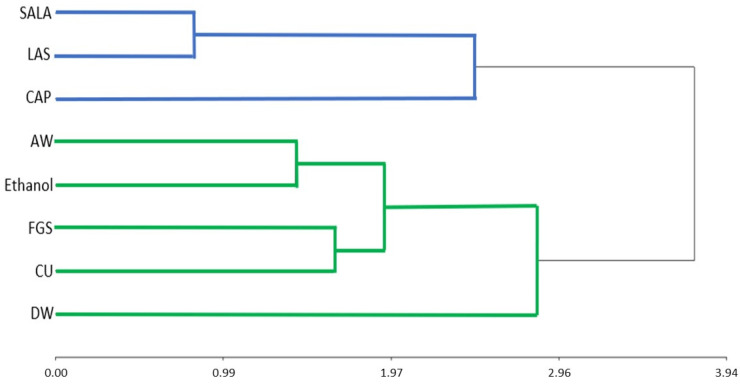
Hierarchical cluster analysis. DW: distilled water, AW: Acetone: water, NaDES: LAS, CAP, FGS, SALA, CU.

The main component analysis (Figure 7) shows an 84% total variability explained by factors F1 and F2. F1 explains 61.1% of the variability and is positively influenced by H_2_O_2_, ABTS, and TA. F2 explains 22.9% of the variability and is influenced by TPC. These components clearly separate SALA, CAP, and LAS from DW, AW, Ethanol 70°, CU, and FGS. The former group of NaDES based on acids has low ABTS activity and a high concentration of TPC. The distance between two assays presents their proximity level; the closer the two vectors are the more significant correlation. For instance, the distance of both ABTS scavenging activity and TPC was very short, so it contained significant positive correlations with similar distances to those between H_2_O_2_ scavenging activity and TF and TA. This information is also supported by a hierarchical clusters analysis that groups together SALAS, LAS, and CAP NaDES (Figure 8).

## 3. Materials and Methods

### 3.1. Reagents

Sucrose, H_2_O_2_, ethanol, sodium phosphate monobasic, sodium phosphate dibasic, and sodium carbonate (Cicarelli, Santa Fe, Argentina), lactic acid (Cicarelli, Origin in France, packed in Santa Fe, Argentina), fructose and acetone (Cicarelli, Origin in USA, packed in Santa Fe, Argentina), glucose (Anedra, Nantong, China), choline chloride (Sigma-Aldrich, Beijing, China), urea, ABTS, 4-aminoantipyrine, bromothymol Blue, quercetin, Folin and Ciocalteu′s phenol reagent, and gallic acid (Sigma Aldrich, St. Louis, USA), citric acid (Anedra, Bs As, Argentina), propylene glycol (Biopack, CABA, Argentina), aluminum chloride (Sigma-Aldrich, Taufkirchen, Germany), chloroform (Cicarelli, Origin in the Republic of Korea, packed in Santa Fe, Argentina), apomorphine hydrochloride (Merck, Darmstadt, Germany), phenol (Cicarelli, Origin in USA, packed in Santa Fe, Argentina), sulfuric acid (Cicarelli, Origin in Spain, packed in Santa Fe, Argentina).

### 3.2. Plant Material

Apical fresh leaves of *Nicotiana tabacum* Var. Virginia (or flue-cured, seed 493) discarded during pre-harvest were collected from crop fields in the town of Perico, Jujuy, Argentina (26°18′ S, 65°37′ W, 1700 m.a.s.l.). The samples were dried at 60 °C, up to constant weight in a forced-air stove and conserved in vacuum-sealed bags until use.

Fresh leaves for histological assays were collected from five random individuals, and preserved in FAA (acetic acid, formalin, water, and alcohol 1:2:7:10) for both optical and scanning electron microscopy (SEM) studies.

### 3.3. Powder Obtention by Milling

The leaves were dried at 60 °C at constant weight in a forced air stove and grounded in a Helix mill (Metvisa^®^, Mod MP-200-Power ½ HP-0.75 Kw, Brusque, Brazil) to obtain leaf powder. The particle size or granulometry was determined by vibrating screen sieving (Zonytest, Buenos Aires, Argentina) with sieves of 840, 500, 149, 105, and 74 µm.

### 3.4. Histological Analysis

#### 3.4.1. Light Microscopy

Both the characterization of the leaf epidermis and the analysis of the fragments in the leaf waste ground fractions were made by using the diaphonization technique according to Dizeo de Strittmater (1973) [44]. The analysis of the structures was carried out through transverse “free hand” sections in the mid portion of the leaf, which was mounted on a dental wax support, and subsequently sectioned with a Microm HM315 rotary microtome (GMI Inc., Ramsey, MN, USA) [45]. The diaphonized tissues were directly observed under the microscope and stained with crystal violet, in the case of powder. The sections were stained with astra blue-safranin and mounted in water-glycerin (1:1) [46,47]. The observations were made with a stereoscopic microscope (Olympus SZX7, Tokyo, Japan), and an optical microscope (Carl Zeiss Axiostar Plus, Göttingen, Germany) attached to a photographic camera (Carl Zeiss AxioCam ERc 5s, Oberkochen, Germany).

In the diaphonized leaf blade, the average size of stomata, both in length and width (µm) and their density (mm^2^) were quantified, for n = 15. Their classification was carried out according to Dilcher (1974) [48].

#### 3.4.2. Scanning Electron Microscopy

For scanning electron microscopy (SEM) 4 mm^2^ leaf fragments, with the middle vein and part of the leaf blade, previously fixed in FAA, were dehydrated with a series of alcohols and acetone and submitted to a dryness critical point with liquid CO_2._ Dry fractions of the sieved leaf waste were directly attached to SEM stubs by using a carbon double-adhesive disc. Samples of leaf fragments and fractions of the powder were coated with gold-palladium by using a Fine Coat Ion Sputter JEOL JFC-1100. The observations were made under a scanning electron microscope (Carl Zeiss Supra 55VP, Oberkochen, Germany) from the Integral Center for Electron Microscopy (UNT-CONICET).

### 3.5. NaDES Preparation

The NaDES: sucrose:lactic acid, (LAS); lactic acid:sucrose:distilled water (SALA); citric acid: propylene glycol (CAP); glucose:fructose:sucrose: distilled water, (FGS); choline chloride:urea:distilled water (CU) were prepared by combining the components in the appropriate mole ratios (Table 3), according to the method described by Dai et al. (2013) [27]. They were prepared in a water bath at 40 °C placed on a magnetic stirrer hot plate. Mixing lasted approximately 20 min until stable transparent liquid was formed.

### 3.6. Powder Extraction

Leaf powder was extracted by maceration in ethanol 70°, 1:10 (*w*:*v*) for 30 min shaking at 100 rpm at 25 °C. The extracts were filtered and kept at −20 °C until use. The same procedure and the same ratio between plant material and the solvent was used with distilled water (DW), acetone: distilled water, 1:2 (AW) and the different NaDES (LAS, SALA, CAP, FGS, CU).

### 3.7. Determination of Chemical Composition

#### 3.7.1. Total Polyphenol and Flavonoid Quantification

The different extractives solutions were standardized by the determination of total phenolic compound content according to Singleton et al., 1999 [49]. Different volumes of extracts were mixed with distilled water, Folin–Ciocalteau reagent, and 15.9% sodium carbonate. The mixture was kept for 20 min at room temperature. The blue color developed was read at 765 nm in UV/visible spectrophotometer (Jasco v-630, Thermo Fisher Scientific, Tokyo, Japan). Total flavonoids were measured by a spectrophotometric assay based on aluminum chloride complex formation according to Woisky and Salatino, 1998 [50]. The conventional and non-conventional solvent controls were performed in each determination to scan any possible interference. The determinations were performed in triplicate and the results were expressed as μg of gallic acid equivalent (GAE) per mL (μg GAE/mL) and quercetin equivalents (QE) per mL (μg QE/mL), respectively. The extraction yield of each solvent was determined as the amount of chemical component per mL of extract or kg plant material.

#### 3.7.2. Total Alkaloids

The alkaloid content of different extractive solutions (EW, DW, AW, NaDES) was determined as described by Önal et al. [51]. The conventional and non-conventional solvent controls were performed to determine any possible interference. Different amounts of extracts were mixed with distilled water, Bromothymol Blue Reagent, and chloroform. The absorbance was recorded at 414 nm in UV/visible spectrophotometer (Jasco v-630). Apomorphine hydrochloride was used as the standard. The experiment was performed in triplicate and the results were expressed as equivalent to apomorphine hydrochloride (µg ACE/mL). The extraction yield of each solvent was determined as the content of alkaloids per mL of extract or kg plant material.

#### 3.7.3. Reducing and Total Sugars

Total neutral sugars were determined by using Phenol sulfuric method [52]; different concentrations of extracts were mixed with distilled water, 80% phenol, and finally concentrated sulfuric acid. It was shaken vigorously in a vortex and placed in a 100° water bath for 20 min. Then, the absorbance at 490 nm was measured in an UV/visible spectrophotometer. Reducing sugars were measured by the Somogyi–Nelson method [53,54], and different amounts of extracts were mixed with Somogyi’s reagent and heated at 100 °C for 15 min. After this time, Nelson’s reagent was added. The absorbance was recorded at 520 nm in UV/visible spectrophotometer (Jasco v-630). The solvent controls were performed to determine any possible interference. The experiment was performed in triplicate and the results were expressed as equivalent of glucose (mg GE/mL). The extraction yield of each solvent was determined as the amount of glucose by mL of extract or kg plant material.

### 3.8. Antioxidant Activity

#### 3.8.1. ABTS Free Radical Scavenging Activity

The antioxidant capacity assay was carried out by the improved ABTS^•+^ spectrophotometric method [55]. In this method, ABTS^•+^ solution was mixed with different amounts of the extractive solution obtained from *N. tabacum* leaf powder. The extracts obtained with non-conventional solvents, as well as each one of the NaDES used, were diluted previously in phosphate buffer (PBS). The solvent controls were also performed to determine any possible interference. Absorbance was recorded at 734 nm in UV/visible spectrophotometer (Jasco v-630) after 6 min. Results are expressed as SC_50_ values. SC_50_ (μg GAE/mL) was defined as the concentration of phenolic compounds necessary to scavenge 50% of ABTS free radicals. Quercetin was used as a reference compound.

#### 3.8.2. Hydrogen Peroxide (H_2_O_2_) Scavenging

The H_2_O_2_ scavenging was assessed according to Fernando and Soysa [56]. The reaction mixture contained phenol (12 mM), 4-aminoantipyrine (0.5 mM), H_2_O_2_ (0.7 mM), sodium phosphate buffer (84 mM) at pH 7, and different concentrations of the extractive solution obtained from *N. tabacum* leaf powder. The extracts obtained with non-conventional solvents, as well as each one of the NaDES used, were diluted previously in phosphate buffer (PBS). The solvent controls were also performed to determine any possible interference. It was kept at 35 °C for 20 min. Then, horseradish peroxidase (0.1 U/mL) was added, and the mixture was incubated at 37 °C for 30 min. The absorbance was measured at 504 nm in UV/visible spectrophotometer (Jasco v-630). Results are expressed as SC_50_ values in μg GAE/mL. Quercetin was used as a reference compound.

### 3.9. Statistical Analysis

For the statistical analysis of the data, the Tukey test was applied, with a level of significance *p* > 0.05, using the statistical package InfoStat V1.1 [57]. Main component analysis and hierarchical clusters (HCA) using the InfoStat V1.1 [57] were applied. Mean values were used for graphs, where each point on the graph corresponded to the mean value for each value. HCA was performed by calculating the Euclidean distance and considering average link clustering as a method for calculating the distance between clusters. In addition, the data were standardized. The number of groups was determined considering the cut-off line half the Euclidean distance.

## 4. Conclusions

This is the first report on the extraction of bioactives from tobacco leaf waste var. Virginia, by using NaDES. The results obtained confirm that NaDES could be selective regarding some chemical groups present in tobacco pre-harvest waste. Furthermore, the extracts obtained have a good yield of phenolic extraction with antioxidant potential, thus allowing the attainment of natural antioxidants without the application of organic solvents from tobacco waste. NaDES are biodegradable and biocompatible, as well as non-toxic. As a result, the NaDES can be used freely, bypassing the solvent removal problem. For this reason, the application of these tobacco waste extracts directly in formulations, such as cosmetics or phytotherapy, seems promising. Further toxicological studies on antioxidants extracted with NaDES from tobacco leaf waste are required for developing products to be used to promote health care on a commercial scale.

## Figures and Tables

**Table 1:** *Nicotiana tabacum* var. Virginia. Length, width, and stomatal density in leaf epidermis.

**Stomata**	**Length** **(µm)**	**Width** **(µm)**	**Density** **(mm^2^)**
Adaxial epidermis	39.00 ± 1.42 ^a^	28.75 ± 0.73 ^a^	23.25 ± 1.45 ^a^
Abaxial epidermis	42.00 ± 1.42 ^a^	32.25 ± 0.73 ^b^	49.25 ± 1.45 ^b^

Different letters in the same column indicate significant differences according to Tukey’s test (*p* ≤ 0.05).

**Table 2:** *Nicotiana tabacum* var. Virginia. Sieved fractions from ground leaf waste.

Mesh Size (µm)	Weight after Sieving (g)	Yield in Percentage (%)
840	4.98	51.98
500	1.88	19.62
149	2.17	22.65
105	0.45	4.70
74	0.08	0.89
<74	0.01	0.15

**Table 3:** Natural deep eutectic solvents (NaDES) used for extraction.

NaDES Code	Components	Molar Ratio	Aspect	pH
LAS	sucrose: lactic acid	0.3:1.2	Viscous transparent	4
SALA	sucrose: lactic acid: distilled water	1:5:7	Viscous transparent	1
CAP	citric acid: propylene glycol	1:4	Transparent	5
FGS	glucose: fructose: sucrose: distilled water	1:1:1:11	Viscous transparent	6
CU	choline chloride: urea: distilled water	1:2:1.5	Transparent	6

**Table 4:** Phenolic compounds, total flavonoids, total alkaloids, reducing sugar and total sugar content of *Nicotiana tabacum* var. Virginia leaf waste powder extracts by using conventional and non-conventional solvents.

*N. tabacum* Leaf Powder	TPC µg GAE/mL	TF µg QE/mL	TA µg ACE/mL	RS mg GE/mL	TS mg GE/mL
**Conventional Solvents**					
DW	824.0 ± 19.6 ^a^	14.9 ± 1.8 ^a^	734.0 ± 2.9 ^b^	5.2 ± 0.4 ^e^	7.3 ± 0.5 ^d^
Ethanol 70°	1275.0 ± 14.5 ^b^	151.3 ± 9.1 ^d^	919.2 ± 3.6 ^c^	2.2 ± 0.1 ^c^	5.7 ± 0.2 ^c^
AW	1300.0 ± 3.0 ^b^	136.6 ± 6.3 ^d^	867.5 ± 0.8 ^c^	3.2 ± 0.2 ^d^	7.2 ± 0.4 ^d^
**Non-Conventional Solvents**					
LAS	2169.0 ± 0.5 ^c^	46.4 ± 3.1 ^b^	68.6 ± 0.4 ^a^	0.40 ± 0.01 ^b^	0.14 ± 0.01 ^a^
CAP	963.0 ± 9.0 ^a^	52.2 ± 4.3 ^b^	78.7 ± 6.0 ^a^	0.060 ± 0.001 ^a^	0.020 ± 0.001 ^a^
FGS	1813.0 ± 3.7 ^c^	89.2 ± 7.5 ^c^	637.2 ± 1.2 ^b^	N/D	N/D
SALA	2198.0 ± 6.4 ^c^	80.5 ± 2.5 ^c^	55.0 ± 3.4 ^a^	0.50 ± 0.01 ^b^	0.030 ± 0.001 ^a^
CU	2076.0 ± 3.7 ^c^	134.0 ± 3.2 ^d^	1123.0 ± 7.0 ^d^	N/D	4.7 ± 1.6 ^b^

TPC: total phenolic compounds; TF: total flavonoids; TA: total alkaloids; RS: reducing sugars; TS: total sugars; GAE: gallic acid equivalent; QE: quercetin equivalent; ACE: equivalent apomorphine hydrochloride; GE: glucose equivalent; DW: distilled water; AW: Acetone: water; NaDES: LAS, CAP, FGS, SALA, CU. Values are reported as mean ± standard deviation of triplicates. Different letters in the same column indicate significant differences between extracts according to Tukey’s test (*p* ≤ 0.05).

**Table 5:** Antioxidant activity of *Nicotiana tabacum* var. Virginia leaf waste powder extracts.

*N. tabacum* Leaf	ABTS (SC_50_ µg GAE/mL)	H_2_O_2_ (SC_50_ µg GAE/mL)
**Conventional Extraction Solvents**		
DW	11.5 ± 0.5 ^d^	38.0 ± 2.4 ^e^
Ethanol 70°	8.9 ± 0.5 ^cd^	21.0 ± 1.6 ^d^
AW	5.0 ± 0.1 ^bc^	44.0 ± 2.2 ^g^
**Non-Conventional Solvents**		
LAS	36.0 ± 0.4 ^f^	8.4 ± 0.6 ^b^
CAP	26.0 ± 1.8 ^e^	3.8 ± 0.1 ^a^
FGS	2.9 ± 0.1 ^ab^	52.0 ± 4.3 ^h^
SALA	41.9 ± 0.2 ^g^	8.7 ± 0.1 ^c^
CU	1.60 ± 0.05 ^a^	40.0 ± 3.1 ^f^
Quercetin	1.40 ± 0.0 ^a^	17.3 ± 0.5 ^b^

SC_50_: Concentration of extracts needed to scavenge 50% of ABTS or H_2_O_2_; GAE: gallic acid equivalent; QE: quercetin equivalent; ACE: apomorphine hydrochloride equivalent; GE: glucose equivalent; DW: distilled water; AW: acetone: water; NaDES: LAS, CAP, FGS, SALA, CU. Values are reported as mean ± standard deviation of triplicates. Different letters in the same column for each extract indicate significant differences according to Tukey’s test (*p* ≤ 0.05).

**Table 6:** Pearson’s correlation coefficients of ABTS and H_2_O_2_ scavenging versus TPC, TF, and TA.

	ABTS	H_2_O_2_
TPC	0.32 *	−0.04
TF	−0.45 *	0.26 *
TA	−0.91 *	0.77 *

* Correlation is significant at *p* < 0.05.

## Data Availability

Not applicable.

## Sample Availability

Samples are available from the authors.
